# Pancreatic cancer immunotherapy biomarkers: from traditional markers to multimodal integration and dynamic monitoring

**DOI:** 10.3389/fimmu.2026.1686658

**Published:** 2026-03-19

**Authors:** Wei-Yi Zhao, Jin-Wei Zhao

**Affiliations:** 1 Department of Hepatopancreatobiliary Surgery of Second Hospital of Jilin University, Jilin University, Changchun, China; 2 Department of Clinical Pathology of Yanbian Hospital of Yanbian University, Yanbian University, Yanji, China

**Keywords:** artificial intelligence, biomarkers, immunotherapy, multimodal integration, nanotechnology, pancreatic cancer

## Abstract

Pancreatic ductal adenocarcinoma (PDAC) remains an intractable cancer marked by delayed diagnosis, rapid progression, and significant resistance to current treatments. Conventional biomarkers, such as CA19-9, have insufficient sensitivity and specificity. Meanwhile, the practical use of newer markers such as the tumor mutational burden and microsatellite instability is limited by the absence of standardized testing protocols and definitive threshold values. Circulating tumor DNA and exosomal miRNA hold promise for continuously tracking tumor dynamics and effectiveness of immunotherapy, but additional validation is necessary before their routine clinical application. Recent advancements in multiomics, nanotechnology, and artificial intelligence have opened new possibilities for more accurate and comprehensive biomarkers. For instance, Shah et al. developed shortwave-infrared-emitting nanoprobes to specifically target CD8^+^ cytotoxic T cells, permitting high-sensitivity *in vivo* imaging in breast cancer models. Batool et al. utilized nanoplasmonic sensors to detect changes in serum programmed death-ligand 1 and cytokine levels within 1–2 weeks post-treatment, achieving picomolar sensitivity. Chang et al. combined fluorescence and photoacoustic imaging in the NanoTrackThera platform, facilitating the real-time monitoring of immunotherapy efficacy. This review highlights the evolution of PDAC biomarkers from traditional markers to multimodal integration and dynamic monitoring. The limitations of current markers and potential of emerging technologies, including metabolic reprogramming markers, epigenetic regulators, and AI-driven predictive models, are discussed. Future directions include multicenter prospective trials to validate multimodal models, standardize detection methods, and increase interdisciplinary collaboration. By integrating genomic, epigenetic, metabolic, and microbiome data, these models can better capture the complexity of PDAC, thereby improving patient outcomes through precision immunotherapy.

## 1 Introduction

Pancreatic ductal adenocarcinoma (PDAC) represents roughly 90% of all pancreatic neoplasms. Currently, PDAC is the seventh most frequent cause of cancer-associated mortality globally. The incidence of PDAC steeply increases with the Human Development Index: age-standardized rates exceed 8 per 100,000 in North America and Western Europe, nearly 5-fold higher than that reported in Sub-Saharan Africa (1–3). Mathematical models predict that, if current trends persist, PDAC will overtake colorectal cancer as the second most lethal malignancy in high-income countries within the next decade (1). These grim statistics are driven by late detection, as more than 80% of patients are diagnosed with locally advanced or metastatic disease, which often limits treatment options to palliative care, resulting in a 5-year survival rate of approximately 3% (4).

**Figure 1:**
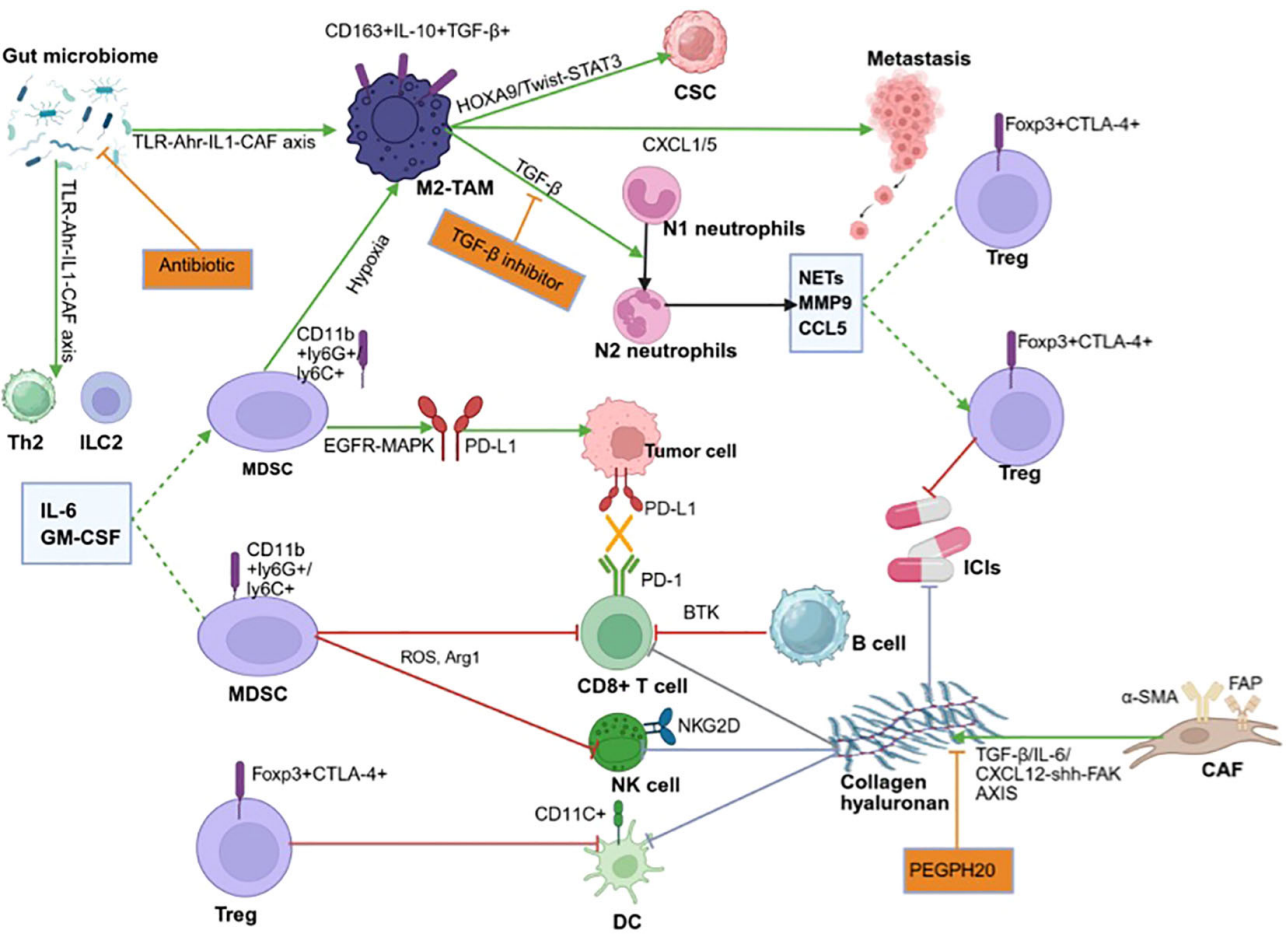
Pancreatic cancer immune microenvironment.

Against this backdrop, immunotherapy, including immune checkpoint blockade (ICB) agents, adoptive T-cell transfer therapy, and personalized neo-antigen vaccines, has completely shifted the treatment landscape for several malignancies, such as lymphoma, melanoma, renal cell carcinoma, and non-small cell lung cancer (NSCLC) (5–8). However, PDAC remains stubbornly refractory. Monotherapy using anti-programmed cell death protein 1 (PD-1), anti-programmed death ligand 1 (PD-L1), or anti-cytotoxic t-lymphocyte-associated protein 4 (CTLA-4) agents, as well as approved combinations such as nivolumab plus ipilimumab, has produced objective response rates lower than 10% and no clear survival advantage (9, 10). These disappointing outcomes are exemplified by several phase III trials, including the PA.7 study (nivolumab plus gemcitabine/nab-paclitaxel vs. chemotherapy alone), which failed to improve overall survival in metastatic PDAC (10), and the CheckPAC trial combining nivolumab/ipilimumab with stereotactic body radiotherapy also demonstrated limited efficacy in refractory disease (2). The root cause lies in a uniquely hostile tumor microenvironment (TME) that is simultaneously desmoplastic, hypoxic, and profoundly immunosuppressive (**Figure 1**), (11–13).

Although PDAC’s immune-desert phenotype is driven by MDSCs, TAMs and CAFs (11–13), **Figure 1**), these same circuits offer druggable markers. ARG1 and ROS released by MDSCs have been explored as metabolic read-outs (14, 15), whereas CAF-enriched FAP and CXCL12 can be quantified by single-cell RNA-seq or plasma-exosome proteomics (16, 17). Yet conventional predictors—PD-L1, TMB, MSI—are rarely positive in PDAC (PD-L1 < 15%, MSI-H < 2%) (18, 19), underscoring the need for technology-enabled alternatives.

Because this self-reinforcing immune escape network undercuts conventional ICB, reliable biomarkers are urgently needed to identify the patients most likely to respond to immunotherapy. Traditional predictors, such as PD-L1 positivity, the tumor mutational burden (TMB), and microsatellite instability (MSI), have poor accuracy in PDAC. PD-L1 is judged by IHC, yet clone choice (22C3, 28-8) and cut-offs (CPS ≥1 vs ≥10) give shifting results (20). Likewise, TMB measured by whole-exome sequencing (WES) shows PDAC’s TMB sits at only 1–1.7 mutations per megabase (mut/Mb), about one-tenth that of melanoma (>10mut/Mb) (21, 22). Emerging data suggest that arginine metabolism defects, characterized by suppressed ASS1 expression, could serve as a companion biomarker for arginine depletion strategies combined with ICB (23). Claudin-18.2 CAR-T also needs pre-screening, since fewer than 30% of tumors express the target (24). Beyond tissue-based biomarkers, non-invasive monitoring modalities offer complementary insights that circumvent the constraints of invasive biopsies and intratumoral heterogeneity.

Liquid biopsy technologies are poised to fill this void. Methylation patterns in blood circulating tumor DNA (ctDNA)—like NDRG2 or PROX1 promoters—can be read with digital PCR or nanopore sequencing down to 0.01% VAF (25, 26). Exosomal miRNAs such as miR-21-5p and miR-1246 are picked up one molecule at a time using nano-plasmonic sensors (27–29). Stool 16S rRNA surveys show that an overgrowth of Gammaproteobacteria links to PDAC’s immune-cold profile, and their lipopolysaccharide (LPS) can be tracked in serum with the LAL assay, giving an indirect microbe-immune read-out (30). However, none of these modalities alone has reached prospective validation. Work in pan-cancer cohorts hints that random-forest or XGBoost models that blend genomic, transcriptomic and imaging traits can push immunotherapy-response AUC above 0.9 (31). Such systems could parse the complex PDAC immune ecosystem, identify latent responders, and monitor adaptive resistance in real time.

In summary, PDAC’s uniquely immunosuppressive TME and the inadequacy of current biomarkers mandate a paradigm shift toward multimodal, artificial intelligence (AI)-driven predictive models (**Table 1**). Rigorous prospective trials are required to translate these insights into precision immunotherapy strategies that can improve the mortality curve of this recalcitrant disease.

**Table 1:** PDAC immune-evasion landscape: current gaps, emerging biomarkers and future directions.

Problem/ Current situation	Conventional predictors & their limitations	Emerging biomarkers/ technologies	Key technical details/ performance	Next steps/ prospects
PDAC immune-desert phenotype driven by MDSCs, TAMs & CAFs, yet itself druggable	Standard ICB biomarkers (PD-L1, TMB, MSI) rarely positive: PD-L1 <15 %, MSI-H <2 %	TME secreted molecules: ARG1, ROS ; FAP, CXCL12.	Detectable by single-cell RNA-seq or plasma-exosome proteomics	Turn immunosuppressive “villains” into quantifiable drug targets
Unstable PD-L1 IHC	Antibody clones (22C3 vs 28-8) and cut-offs differ, yielding shifting results	Replace IHC with liquid biopsy or multi-omics models	Digital PCR, nanopore sequencing, machine-learning integration	Reduce sampling heterogeneity and improve reproducibility
Low TMB	WES shows PDAC only 1–1.7 mut/Mb, failing to enrich ICB responders	Metabolic-defect marker: ↓ASS1 expression → arginine-metabolism defect	Pair arginine-depletion + ICB combination	“Heat up” cold tumors through metabolic intervention
Scarce targets	Claudin-18.2 positive in <30 % of tumors, requiring pre-screening	Claudin-18.2 CAR-T pre-screening	IHC or RNA-scope quantification	Increase cell-therapy enrollment efficiency
No single analyte prospectively validated	Above traditional metrics achieve low overall AUC	Multi-modal liquid biopsy: • ctDNA methylation (NDRG2, PROX1 promoters) • exosomal miRNAs (miR-21-5p, miR-1246) • gut-microbiota LPS indirect immune read-out	• Digital PCR / nanopore → 0.01 % VAF • nano-plasmonic sensors single-molecule detection • stool 16S + serum LAL assay	Technologies await prospective trials; large PDAC cohorts needed
Insufficient data integration	Single-dimensional biomarkers rarely exceed AUC 0.7–0.8	Machine-learning ensemble: random-forest / XGBoost integrating genomic + transcriptomic + imaging features	Pan-cancer studies already show AUC >0.9	Build PDAC-specific multi-omics predictor for real-time monitoring and adaptive-resistance tracking

**Figure 2:**
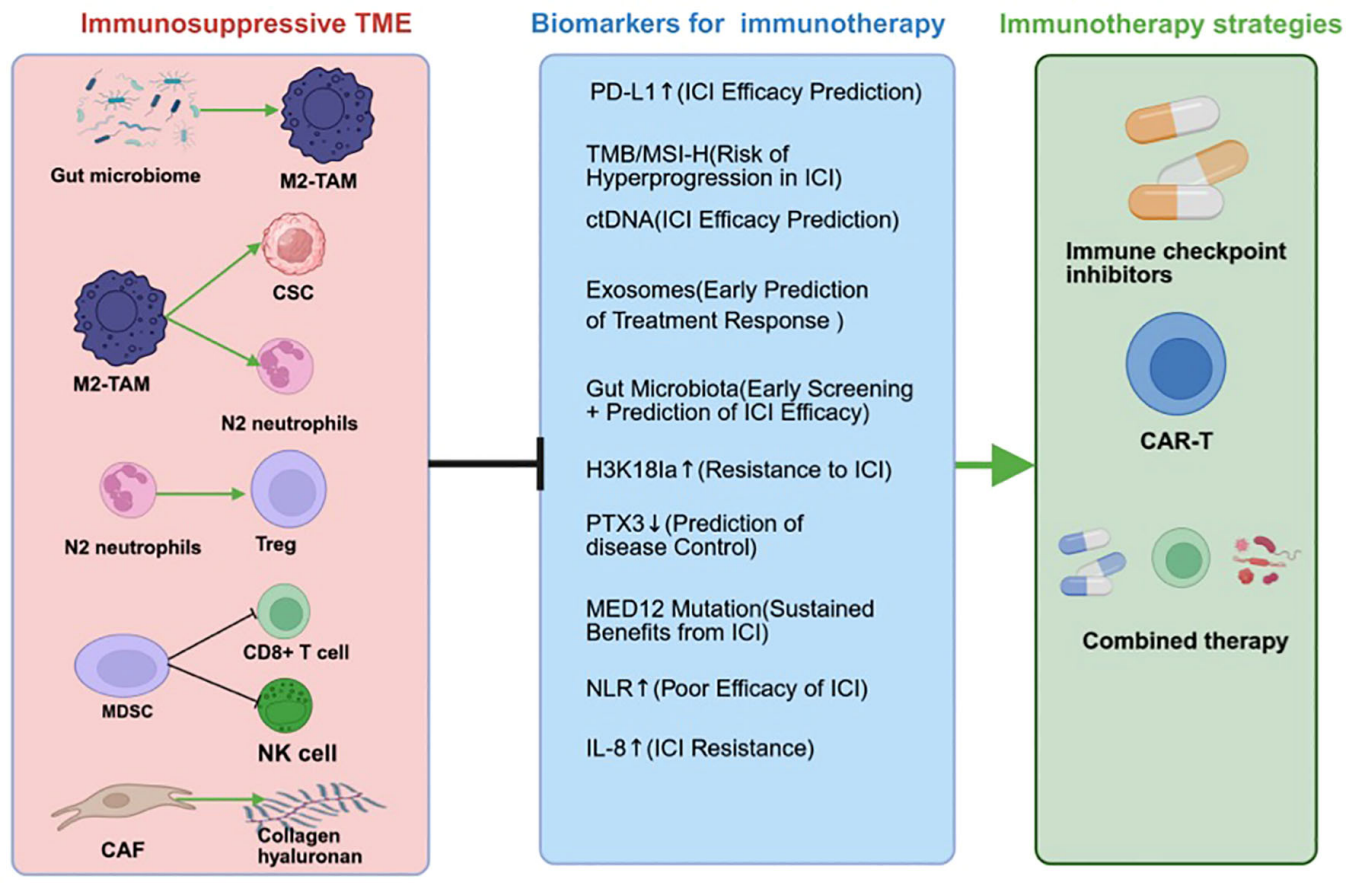
Pancreatic cancer immunotherapy biomarkers and their roles.

This review systematically summarizes the latest advances in biomarker research for PDAC immunotherapy, focusing on traditional and emerging biomarkers, their limitations, and the potential of liquid biopsy and multiomic integration (**Table 2**). This review aims to provide a comprehensive overview of the current state of biomarker research in PDAC immunotherapy and highlight the need for multimodal, AI-driven predictive models (as illustrated in **Figure 2**). This article intends to provide valuable insights for oncologists, researchers, and clinicians involved in the treatment and study of pancreatic cancer, guiding future research directions and clinical practice.

**Table 2:** Summary of pancreatic cancer immunotherapy biomarkers.

Category	Markers	Testing methods	Clinical value (related to immunotherapy)	Advantages	Limitations/issues to be resolved
Traditional serum protein	CA19-9	Chemiluminescence/ELISA (serum)	Dynamic monitoring of treatment response; the extent of the decline is related to the survival benefit.	Technologically mature and low cost.	Low specificity (can also be elevated in cholangitis); false negatives in Lewis-negative populations.
Exosomes	Exosomal miRNA	RT-qPCR (serum, pancreatic fluid, saliva exosomes)	Early prediction of treatment response (within 2 weeks); can monitor relapse.	Non-invasive, repeatable; reflects the immune suppression status of the TME.	Separation standards are not unified; there is a lack of consensus on thresholds and quality control.
Immune checkpoint	PD-L1	IHC 22C3/28-8 (tumor tissue or TILs); flow cytometry/IF	Traditional companion diagnostics have limited predictive efficacy when used alone; however, combining them with TMB and MSI can improve accuracy.	Technologically mature, FDA-approved.	Tumor heterogeneity is high; dynamic expression; the positivity rate is low in PDAC (13%–64%).
Genomic instability	TMB	NGS (tissue or ctDNA)	Patients with TMB-H (≥10 Mut/Mb) have better responses to ICIs.	Highly overlaps with MSI/dMMR; a single test covers the entire genome.	The proportion of TMB-H tumors in PDAC is only approximately 1.1%; the threshold and panel have not been standardized.
	MSI/dMMR	NGS/PCR (tissue); liquid biopsy Guardant360	The ORRs of ICIs in patients with MSI-H tumors can reach 77%.	Liquid biopsy for dynamic monitoring; FDA approval granted for indications without tumor type restrictions.	In PDAC, only 2% of tumors are MSI-H; high-depth sequencing is needed to ensure sensitivity.
Metabolic reprogramming	H3K18 lactylation (H3K18la)	ChIP-seq/mass spectrometry (tissue); ELISA (serum lactate)	High expression indicates immune escape and ICI resistance; combined inhibition of glycolysis can improve efficacy.	First revealed the metabolic/epigenetic/immune axis, with potential for druggability.	Clinical thresholds have not yet been established; there is a lack of methods for monitoring dynamic changes.
Metabolic indicators	MPI (nine-gene model including ANLN, PKMYT1 and HMGA1)	NGS expression profile	High-risk groups have an immunosuppressed TME and resistance to ICI, which can guide targeted drug selection.	Integrating multiple genes is more robust than a single gene.	External large sample validation is required; threshold standardization remains to be determined.
Immune regulatory protein	PTX3	ELISA (serum)	Dynamic fluctuations in levels can predict disease control, with better accuracy than CA19-9.	Easy to operate; STARPAC tests have been validated.	Prospective phase III studies are needed to confirm the cutoff.
Epigenetic regulation	MED12 mutation	NGS (WES/MSK-IMPACT)	Patients with mutant tumors experience sustained benefits and prolonged OS from ICIs; mutations lead to an immune “hot” TME.	Its utility has been confirmed in both pan-cancer models and mouse PDAC models.	Mutation frequency is low; functional experiments and clinical data need to be expanded.
lncRNA	m6A-/copper death-/methylation-related lncRNA signature	NGS expression profile	Risk scores can predict the benefits and prognosis of immunotherapy.	It can simultaneously reflect the mutation burden and immune microenvironment.	The model is complex; it needs to be simplified to the qPCR or ddPCR platform.
Liquid biopsy dynamic marker	ctDNA (*e.g.*, KRAS mutations, HOXD8/POU4F1, BRCA/ATM methylation)	ddPCR/NGS (plasma)	A low baseline level or early decline indicates the effectiveness of ICI therapy or chemotherapy; post-operative ctDNA positivity suggests a high risk of recurrence.	Non-invasive, real-time; detects progression 23 days earlier than imaging techniques.	Sensitivity is affected by the tumor burden; clonal hematopoiesis interference.
Liquid biopsy dynamic marker	CTCs (Φ)	Microfluidic enrichment + immunofluorescence	The decrease in Φ is associated with the response to PARP inhibitors; can predict efficacy in CA19-9–negative patients.	Single-cell sequencing can capture rare subpopulations.	The technology is complex; standardization and quality control need improvement.
Host microecology	Intestinal microbiota (27-strain/30-strain model)	Metagenomic sequencing (feces)	Early screening + predicting ICI efficacy; specific bacteria associated with chemotherapy resistance.	Non-invasive; cross-ethnic validation	Antibiotics and dietary interference are significant; mechanisms and intervention strategies need further exploration.
Systemic inflammation	NLR, dNLR, PLR, SII	Complete blood count	Baseline high values or increases during treatment suggest poor efficacy of ICI and shorter OS.	Low-cost, real-time dynamic monitoring	Influenced by factors such as infection and hormones, a multiple-time point joint analysis is needed.
Systemic inflammation	IL-8	ELISA (serum)	High levels are associated with ICI resistance and rapid progression; a decrease suggests treatment response.	Validated in various tumors	There is currently no unified cut-off; prospective trials need to incorporate multivariable models.
Single-cell multi-omics	csCAF gene signature (C3, CFD, CSF1); basal-like vs classical sub-clone maps; JUNB/AP1 axis	scRNA-seq + scATAC-seq (+ Visium HD for spatial)	Identify immune-exhaustion drivers; predict ICB benefit (anti-PD-1 response ↑ from 12 % → 58 % after JUNB/AP1 blockade in KPC)	One integrated run gives epigenome + transcriptome + immune receptors; near-single-cell spatial overlay	Fresh-tissue dependency; high cost; bioinformatic complexity; needs prospective clinical validation outside KPC model
Ultrasensitive detection	Serum PD-L1 (0.3 pg mL^-^¹ LOD); KRAS G12D ctDNA (0.0003 % VAF); circRNA/miRNA (1 aM); CD8-targeted SWIR probe	Gold nano-island SPR chip; NAPTUNE dual-enzyme cascade; short-wave IR imaging	Early on-treatment response read-out (92 % match for PD-L1 drop at day 14); detect minimal residual disease; image CTL infiltration (250 µm resolution)	30–45 min assay time; no PCR amplification; attomolar LOD; 95 % sensitivity, 100 % specificity for KRAS G12D	Standardization across centres; reagent batch variability; limited commercial kits; need large PDAC-specific response trials
AI-integrated predictive models	CT/MRI radiomic score; CA19-9 slope; ctDNA decay constant k; IL-8 % change; SHAP/Grad-CAM features	Contrast/non-contrast CT or MRI + routine blood panels + federated-learning hub (PDAC-IMMUNO-AI)	Non-invasively select ICB beneficiaries (AUC 0.79–0.89); real-time model refresh every 5 min; define low-risk group with mortality HR 0.16 and ORR 65 % vs 33 %	Leverages existing clinical data; explainable AI; federated privacy protection; outperforms TMB (0.58) & PD-L1 IHC (0.54)	Model locked mainly on NSCLC data; PDAC-specific trial (NCT05678921) ongoing; requires multi-centre data harmonization; regulatory pathway for dynamic algorithms unclear

## 2 Traditional biomarkers and their limitations

### 2.1 Conventional serum marker CA19-9

CA19-9, a sialylated Lewis A glycan generated by aberrant glycosylation in PDAC (32), remains the most widely used serum biomarker for diagnosis, resectability assessment, and prognosis (33, 34). Preoperative CA19-9 > 1000 U/mL predicts unresectability with 85% specificity, whereas a 50% decline in CA19–9 levels after multidrug neoadjuvant therapy was associated with median overall survival (OS) of 26 months, versus 15 months for smaller declines in CA19–9 levels (P = 0.02) (33, 34). In the immunotherapy setting, serial CA19–9 kinetics mirror radiographic responses: patients achieving levels ≤ 37 U/mL at 12 weeks on PD-1/CTLA-4 blockade exhibit significantly longer progression-free survival (PFS) than those with higher levels (8.4 months vs. 3.1 months; hazard ratio [HR] = 0.42). In addition, OS was 16.7 months for patients with CA19–9 levels of ≤37 U/mL, compared with 7.9 months for those whose levels remained elevated (HR = 0.38) (35). Mechanistically, CA19–9 binds to EGFR, triggering downstream MAPK signaling, promoting the recruitment of MDSCs. Antibody-mediated CA19–9 blockade restores CD8^+^ T cell infiltration and synergizes with checkpoint inhibitors in murine PDAC models (36, 37). Beyond PDAC, Du et al. reported that post-treatment CA19–9 increases of >20% from baseline predict early progression in patients with hepatocellular carcinoma receiving PD-1 inhibitors (38), whereas Wang et al. demonstrated that maintaining CA19–9 levels at ≤37 U/mL after 12 weeks of pembrolizumab-based therapy in advanced gastric cancer conferred superior response rates (63% vs. 28%, *P* < 0.01) and extended PFS/OS (35). Nevertheless, CA19–9 has limitations: 79%–81% sensitivity for symptomatic PDAC, missing small or early-stage tumors; 5%–10% of Lewis-negative individuals cannot synthesize CA19-9, yielding false negatives; and benign hepatobiliary disorders (*e.g.*, cholangitis, cholestasis) elevate CA19–9 levels, reducing specificity to 80%–90% (34, 39). Serial CA19–9 monitoring combined with ctDNA kinetics and inflammatory indices (*e.g.*, neutrophil-to-lymphocyte ratio [NLR], interleukin IL-8) might augment its predictive accuracy and guide adaptive immunotherapy strategies (34, 39).While serum markers like CA19–9 provide systemic disease monitoring, exosome-based biomarkers offer deeper insights into tumor-immune microenvironment interactions through their unique cargo delivery mechanisms.

### 2.2 Exosomal miRNA

Exosomes are nanoscale vesicles that deliver functional cargo and mediate intercellular communication. The miRNA content of exosomes critically shapes tumor immunity and enables non-invasive liquid biopsy for PDAC (40). Tumor-derived exosomes carrying miR-301a or miR-21 silence PTEN, activate PI3K/AKT, and polarize macrophages toward the immunosuppressive M2 phenotype, promoting angiogenesis (41). Exosomal miR-21 and miR-10b also blunt CD8^+^ T cell cytotoxicity by activating the Ras/ERK pathway or repressing PTEN, driving T-cell exhaustion (42). Moreover, exosomes transfer PD-L1 mRNA or miRNAs that induce PD-L1 expression in recipient cells, expanding the area of immune evasion (43–45). In PDAC, exosomal miRNAs forecast treatment efficacy before changes are detected by imaging. miR-21 and let-7g-3p levels predict response within two weeks of therapy initiation with 89% sensitivity (46). *In vitro*, CAFs overexpressing miR-21 secrete MMP-3, MMP-9, PDGF, and CCL-7, enhancing PDAC cell invasion and gemcitabine resistance *via* PDCD4 downregulation (47). *In vivo*, CAF-derived miR-21 promotes desmoplasia and chemoresistance, as miR-21 suppression attenuates fibrosis and restores drug sensitivity (47).

Noninvasive detection is feasible using blood, pancreatic juice, or saliva. Que et al. (48) used RT-PCR to quantify four miRNAs in serum exosomes: miR-17-5p, miR-21, miR-155, and miR-196a. High miR-17-5p levels were correlated with metastasis and advanced stage. Meanwhile, miR-21 levels were higher in patients with PDAC than in healthy controls or patients with chronic pancreatitis, but miR-21 levels were not correlated with differentiation or stage, suggesting complementary diagnostic value. Lai et al. (49) compared exosomal profiles among PDAC, chronic pancreatitis, and healthy groups. Elevated miR-10b, miR-21, miR-30c, and miR-181a accurately distinguished the PDAC group from the two other groups. The levels of these miRNAs were normalized within 24 h after tumor resection. Notably, all 29 patients with PDAC exhibited increased miR-10b and miR-30c levels, whereas only eight displayed normal or only mildly elevated CA19–9 levels, indicating the superiority of these miRNAs over traditional markers such as GPC1 and plasma CA19-9. Additionally, miR-191, miR-21, and miR-451a are upregulated in PDAC and intraductal papillary mucinous neoplasm (50). Researchers have successfully isolated these exosomal miRNAs from plasma, pancreatic juice, and saliva, underscoring their versatility as peripheral biomarkers (29, 51).

Beyond diagnosis, exosomal miRNAs can inform prognosis. Plasma exosomal miR-451a predicts disease recurrence (52). Kawamura et al. (53) analyzed portal vein exosomes and identified correlations of miR-4525, miR-451a, and miR-21 with post-operative recurrence and OS, providing surgeons with actionable risk stratification.

Interestingly, exosomal miRNAs have been employed as biomarkers for the immune treatment response in pancreatic cancer. One study assessed the serum miR profiles, including miR-21, miR-210, miR-221, and miR-7, of patients with PDAC undergoing lapatinib and capecitabine therapy. Of 17 patients, six patients were classified as non-responders, whereas another six patients who completed more than four cycles of treatment were identified as responders. The average OS for non-responders was 6.5 months, versus 10.4 months for responders. A significant increase in serum miRNA levels was observed early in non-responders, suggesting that elevated levels of specific serum miRNAs are linked to resistance to lapatinib and capecitabine (54).

miR-155, located on human chromosome 21, is upregulated by the activated K-Ras gene (55), and it is highly expressed in most patients with pancreatic cancer, with its overexpression becoming more frequent as the disease progresses. miR-155 plays key roles in tumorigenesis and progression by inactivating the tumor suppressor gene p53 through TP53INP1 suppression (56). High miR-155 expression confers resistance to gemcitabine through its anti-apoptotic effects and further promotes the release of exosomes containing miR-155, further establishing gemcitabine resistance in tumor cells. Thus, blocking exosome transport can reduce gemcitabine resistance caused by miR-155 (55).

In summary, exosomal miRNAs are key nodes in pancreatic cancer treatment through their ability to regulate the immune microenvironment. Their clinical translation requires interdisciplinary collaboration to overcome technical bottlenecks, and engineered delivery and dynamic monitoring strategies have displayed potential for reshaping therapeutic paradigms (57).Beyond circulating biomarkers, tissue-based expression of immune checkpoint proteins provides direct assessment of the tumor’s immune evasion capacity at the cellular level.

### 2.3 Immune checkpoint marker PD-L1

PD-L1 expression patterns differ starkly across malignancies. In NSCLC, tumor proportion score (TPS) ≥ 50% is strongly linked to immune evasion, and it is a strong indicator of robust responses to PD-1/PD-L1 blockade (58, 59). Comparable associations have been reported in breast cancer, colorectal cancer, melanoma, and ovarian cancer, in which PD-L1 positivity is correlated with prognosis and improved outcomes under checkpoint inhibition (60–63).

Conversely, published data indicate tumor cell PD-L1 positivity rates ranging from 13.5% to 64%, and PD-L1 positivity is associated with heightened tumor aggressiveness, an immunosuppressive microenvironment, and diminished OS. Importantly, PD-L1 is present in cancer cells, and it is also highly expressed in TAMs, immunosuppressive myeloid cells such as MDSCs, and Tregs within the TME, collectively inhibiting cytotoxic T cell responses *via* the PD-1/PD-L1 axis (64).

Immunohistochemistry (IHC) remains the standard method for assessing PD-L1 expression. Formalin-fixed tumor sections are scored for membranous staining, and the result is both a biomarker for anti-PD-L1 therapy and an indicator of OS (65). In a focused study of 13 resected PDAC specimens, Zheng et al. observed co-overexpression of human mixed-lineage leukemia protein-1 and PD-L1. PD-L1 was detected in 60%–90% of cells, localizing to both the cell membrane and cytoplasm (64). Flow cytometry of 10 PDAC cell lines further revealed high PD-L1 surface expression in nine cell (64).

lines Despite these data, PD-1/PD-L1 inhibitor monotherapy has consistently displayed limited efficacy against PDAC in clinical trials, with objective responses observed almost exclusively in the 1%–2% of patients whose tumors harbor MSI or deficient mismatch repair (dMMR) (65). Consequently, current guidelines recommend combining PD-L1 IHC with additional biomarkers, namely TMB, dMMR, and MSI, to enhance the accuracy of predicting the benefit of immunotherapy (65).

The use of PD-L1 as a solitary biomarker is further hampered by marked intratumoral heterogeneity. PD-L1 expression can differ between core biopsies and resected specimens and fluctuate under selective pressures such as chemotherapy or radiotherapy (66). Dynamic monitoring, therefore, appears essential, but repeated tissue sampling is often impractical in PDAC. Emerging liquid biopsy approaches, including circulating exosomal PD-L1 mRNA and single-cell RNA sequencing (RNA-seq) of fine-needle aspirates, are being explored to circumvent this limitation (66). Mechanistic studies have additionally provided new therapeutic strategies. One recent report demonstrated that TAM-secreted factors promote the nuclear translocation of pyruvate kinase M2, which transcriptionally upregulates PD-L1, thereby reinforcing the immunosuppressive niche (67). Another investigation identified oncogenic FOXP3 as a direct driver of PD-L1 expression in PDAC cells and demonstrated that dual blockade of PD-L1 and the chemokine CCL5 significantly delayed tumor growth in murine models (68).

Taken together, effective immunotherapy for PDAC requires an integrative strategy that combines the baseline TMB, gene expression signatures, and real-time PD-L1 quantification with dynamic assessments of immune-related markers. Although PD-L1 retains clear but restricted value as a biomarker, its limitations mandate the incorporation of complementary molecular readouts, development of more sensitive detection platforms, and exploration of rational combination regimens to maximize therapeutic efficacy in PDAC.

### 2.4 Genomic instability markers: TMB and MSI

TMB and MSI are two important therapy response biomarkers with potential applicability in assessing the immune response of pancreatic cancer.

#### 2.4.1 TMB

TMB is assessed by evaluating the number of somatic non-synonymous mutations per megabase, and it represents a biomarker for the tumor’s mutational profile and predicts the neoantigen load and responsiveness to immune checkpoint inhibitors (ICIs) (69). In a study of PDAC, Lawlor et al. identified 47 TMB-high (TMB-H) tumors, representing only 1.1% of all patients with PDAC who underwent targeted sequencing (21). These TMB-H lesions were predominantly located in the pancreatic head, and they exhibited distinctive histologic features. Specifically, 14% and 4% were mucinous–colloid and medullary, respectively, frequencies that significantly exceed the findings in TMB-low PDAC, and these findings were closely linked to the MSI/dMMR status (21). Clinically, patients with TMB-H PDAC displayed objective responses to ICIs, suggesting that even this rare molecular subset can benefit from ICB (21).

The findings in Zhu’s real-world cohort corroborate this result. Patients with PDAC and TMB ≥ 10 Mut/Mb who received ICIs experienced significantly longer median OS than historical controls (70). Palmeri et al. expanded this finding beyond PDAC by analyzing 157 patients with advanced solid tumors whose MSI/TMB status was determined by next-generation sequencing (NGS) (71). Among individuals treated with immunotherapy, median PFS reached 24.2 months, significantly higher than that observed in patients receiving chemotherapy (6.75 months; P = 0.042). The approximately 4.7-fold higher PFS strongly favored immunotherapy, underscoring TMB’s potential as a predictive biomarker across tumor types (71). Functional studies further indicated that TMB-H PDAC tumors, relative to microsatellite-stable tumors, mount more robust anti-tumor immunity driven by cytotoxic T cells primed *via* helper T cells and dendritic cells (72).

Despite these encouraging signals, TMB assessment in PDAC faces practical hurdles. The overall mutational load of PDAC is low, as approximately 1.1% of patients achieve TMB-H thresholds (69). Tissue acquisition is challenging, and inter-laboratory variations in sequencing depth, filtering algorithms, and cutoff definitions impede standardization (69). Encouragingly, approximately 60% of patients with TMB-H PDAC also display high MSI (MSI-H) (21). Consequently, a combined MSI/TMB evaluation could more accurately identify patients with PDAC likely to benefit from ICIs while minimizing futile exposure and guiding personalized immunotherapeutic strategies (21).

#### 2.4.2 MSI

MSI arises from defective mismatch repair proteins, and it has been well characterized in colorectal and gastric cancers. However, large consecutive cohort studies revealed that only 2% of PDAC tumors are MSI-H (73). Despite the low incidence, the MSI-H status is a favorable prognostic factor, probably because these tumors elicit a stronger innate anti-tumor immune response (74).

Recent diagnostic advances leverage liquid biopsies to detect MSI through ctDNA, circumventing the difficulty of obtaining adequate pancreatic tissue and permitting serial monitoring. In one illustrative case, ctDNA identified MSI-H PDAC, and the patient achieved a sustained clinical response to pembrolizumab (75). A broader Guardant360 ctDNA registry captured 52 patients with MSI-H PDAC. Among nine patients who received ICIs, the overall response rate (ORR) was 77% according to RECIST, supporting the clinical utility of non-invasive MSI testing (76).

Front-line combination strategies are also being explored. Small prospective series demonstrated that adding pembrolizumab to the FOLFIRINOX regimen improved outcomes among patients with MSI-H PDAC compared with the findings in historical controls treated with chemotherapy alone (77, 78).

Beyond MSI, TMB adds predictive granularity. Palmeri et al. employed FoundationOne CDx NGS to profile the MSI/TMB status in 157 patients with advanced solid tumors. Immunotherapy conferred an ORR of 55.9% and PFS of 24.2 months, versus 6.75 months for chemotherapy (P = 0.042) (71). A focused review identified eight patients with TMB-H PDAC who received anti-PD-1 therapy. Five patients achieved partial responses, one had stable disease for 30 months, and two patients with an MSI/dMMR status achieved a complete response. However, the small sample limited definitive conclusions (21). These data suggest that combining TMB assessment with MSI/dMMR assessment refines responder identification because TMB captures the global mutational load and MSI focuses on mismatch repair-driven mutations (21).

Long-term outcomes from the KEYNOTE-158 basket trial (cohort K) further validated MSI-H/dMMR as a robust selection filter. After a median of 47.4 months of follow-up (n = 355 non-colorectal tumors), pembrolizumab achieved an ORR of 18.2% (4/22) in the pancreatic subgroup and median PFS and OS of 2.3 and 18.0 months, respectively. Notably, remission was maintained for up to 45.5 months in the four responders (79). The durable benefit observed in this molecular subset supports routine MSI and TMB evaluation.

In summary, although fewer than 5% of patients with PDAC have both MSI-H and TMB-H tumors, these markers define a population of patients with meaningful clinical responses to pembrolizumab. Continued optimization of ctDNA-based assays and prospective trials integrating MSI, TMB, and real-time monitoring are needed to expand precision immunotherapy in pancreatic cancer.

## 3 Emerging biomarker research advances

### 3.1 TME-related biomarkers

#### 3.1.1 Metabolic reprogramming biomarkers

Metabolic dysregulation is a hallmark of pancreatic cancer. Pancreatic cancer cells can reprogram their metabolism to meet biosynthesis, energy uptake, and redox demands, thereby sustaining malignant growth. Moreover, pancreatic cancer cells engage in metabolic interactions with cells in the TME, promoting tumor progression and even influencing immune responses. Metabolic reprogramming markers, such as those related to glycolysis, glutamine metabolism, and cholesterol metabolism, play important roles in pancreatic cancer by regulating the TME and immune cell function. Monitoring these markers could help better assess patients’ responses to immunotherapy (80).

##### 3.1.1.1 Metabolic prognostic index

Ma et al. (81) mined RNA-seq data from 178 TCGA-PAAD and 95 CPTAC-PDAC samples to construct a mitochondrial energy metabolism-related gene (MEMG) signature comprising 11 hub genes (*e.g.*, ATP5F1A, NDUFS1, SDHB). Patients were categorized into high- and low-risk groups based on MEMG scores. High MEMG scores were associated with a median OS of 14.3 months, compared with 29.8 months for low MEMG scores (HR = 2.34, 95% confidence interval [CI] = 1.56–3.51, *P* < 0.001). The high-risk subset exhibited an elevated TMB (mean, 4.2 Mut/Mb vs. 2.1 Mut/Mb, *P* = 0.008), reduced CD8^+^ T cell infiltration (median, 28 cells/mm^2^ vs. 95 cells/mm^2^, *P* = 0.005), and higher PD-L1 expression (mean H-score, 172 vs. 61, *P* < 0.001). Drug sensitivity modeling indicated that tumors with high MEMG expression were resistant to gemcitabine (IC_50_, 12.4 µM vs. 6.7 µM) but hypersensitive to the mitochondrial complex I inhibitor IACS-010759 (IC_50_, 0.31 µM vs. 1.8 µM). The MEMG signature thus offers a mitochondria-centered prognostic tool that links metabolic reprogramming to immunosuppression and guides precision therapy in pancreatic adenocarcinoma.

##### 3.1.1.2 Pentrexin 3

PTX3 is a homopentameric protein belonging to the pentraxin superfamily. Previous studies described PTX3 as an evolutionarily conserved fluid-phase pattern recognition molecule involved in innate humoral immunity and inflammatory response regulation (82). Thus, functional analysis of PTX3 in lung adenocarcinoma and its role in the TME is of great importance. Moreover, the sensitivity of tumor cells to anti-cancer agents is significantly correlated with PTX3 expression (83). The plasma concentration of PTX3, a stromal glycoprotein, discriminates PDAC with high accuracy (area under the curve [AUC] = 0.91) and dynamically mirrors tumor–stroma signaling (84). In the WDR5–H3K4me3 epigenetic study by Lu et al., baseline PTX3 levels < 3.2 ng mL^−1^ identified a subset of patients with PDAC whose tumors relied less on PD-L1–mediated immune escape. These patients achieved an ORR of 60% (6/10) following pembrolizumab treatment, whereas only 15% (3/20) of PTX3-high patients (> 7 ng mL^−1^) responded to treatment (85). Serial sampling revealed that a ≥ 35% decline in PTX3 expression after two cycles of combined stromal-targeting therapy plus anti-PD-1 therapy was correlated with prolonged PFS (median, 9.4 months vs. 3.1 months, P = 0.017), underscoring PTX3’s utility for treatment selection and early efficacy monitoring (84, 85). Additionally, Goulart et al. measured plasma PTX3 levels in 478 samples (312 PDAC, 192 benign, 200 healthy). The median level was 8.4 ng/mL in PDAC samples, versus 1.9 ng/mL in healthy control samples (AUC = 0.88, cutoff = 5.2 ng/mL, sensitivity = 79%, specificity = 83%). Post-resection PTX3 expression decreased by 61% within 30 days and rebounded at recurrence, supporting its role as a stromal PDAC biomarker for early detection and relapse monitoring (86).

##### 3.1.1.3 Lactate and its associated histone lactylation modification (H3K18la)

Lactate, as the end-product of tumor glycolysis, has recently been confirmed as a key signaling molecule regulating immune evasion. Li et al. demonstrated the role of a positive feedback loop between glycolysis and H3K18la in PDAC. The study found that elevated H3K18la levels are significantly linked to poor prognosis in patients, indicating a potential target for therapeutic intervention. By inhibiting glycolysis or targeting H3K18la, the cytotoxic function of CD8^+^ T cells can be enhanced, thereby improving the response to immunotherapy. There are similarities in the mechanisms of H3K18la between NSCLC and PDAC. H3K18la promotes immune evasion by triggering the POM121/MYC/PD-L1 pathway. The combination of metabolic reprogramming (such as inhibiting glycolysis) and immunotherapy can reverse this immune evasion (87).

Zhang et al. highlighted the crucial role of histone lactylation (especially H3K18la) in pancreatic cancer. H3K18la levels are significantly increased in pancreatic cancer, resulting in activation of the transcription of the acetyltransferase ACAT2, which promotes cholesterol synthesis and exosome delivery. This process polarizes TAMs toward the immunosuppressive M2 phenotype, forming a lactate/H3K18la/ACAT2/cholesterol immunosuppressive axis. This axis is key to reprogramming the TME, and it is positively linked to resistance to anti-PD-1 therapy (HR = 2.1, P < 0.01) (88). Preclinical studies found that targeting this axis achieved an 80% tumor suppression rate and extended survival by 150%, offering a new strategy to improve the anti-PD-1 response in pancreatic cancer (88). Additionally, researchers developed a PROTAC targeting ACAT2 to augment the efficacy of ICB (89).

The aforementioned studies offer novel therapeutic insights and directions for treatment adjustments for patients with pancreatic cancer and high H3K18la expression, holding promise to increase the effectiveness of immunotherapy and improve patient outcomes.

#### 3.1.2 Epigenetic regulatory biomarkers

##### 3.1.2.1 Mediator subunit 12

MED12, as a crucial regulator of transcription, is a large multisubunit protein complex that serves as a crucial regulator of transcription. Mutations in MED12 can lead to tumorigenesis either through disruption of the MED12–CycC interaction or independent mechanisms. Additionally, mutations in MED12 attenuate CDK8 kinase activity, and they are associated with drug tolerance (90). Pan-cancer whole-exome sequencing (WES, n = 474) and MSKCC validation (n = 1513) revealed that patients harboring mutant MED12 concomitantly with a higher TMB and increased CD8^+^ T cell and dendritic-cell infiltration experienced a superior durable clinical benefit and prolonged PFS and OS under ICI therapy (91). Tang et al. further demonstrated that MED12 deletion in PDAC models unleashed endogenous retrotransposons *via* loss of HP1A-mediated H3K9me3 silencing, triggering cytosolic DNA sensing and type I interferon activation (92). Consequently, MED12-null tumors exhibited a 2.3-fold increase in CD8^+^ T, natural killer, and NK1.1^+^ T cell infiltration and a 1.8-fold improvement in the anti-PD-1 response (92). In human PDAC, MED12-low tumors (n = 63) featured 2.6-fold more retrotransposons, 4.3-fold higher IFN-β expression, and 3.2-fold more CD8^+^ T cells than MED12-high tumors (n = 113). Among 42 ICB-treated patients, MED12-low patients achieved an ORR of 45% (versus 14%, P = 0.018) and longer PFS than MED12-high patients (9.7 months vs. 3.4 months, HR = 0.38) (92). Thus, MED12 mutation or depletion remodels the TME into an inflamed state, highlighting its utility as a predictive biomarker and therapeutic target to overcome immunotherapy resistance in pancreatic cancer.

##### 3.1.2.2 Long non-coding RNA

lncRNAs have risen to prominence as crucial regulators of PDAC biology, orchestrating tumor immunity and dictating responses to immunotherapy (93). Huang et al. (94) examined 178 TCGA-PAAD samples and constructed a seven-lncRNA signature linked to m6A/m5C/m1A methylation. In this cohort, the high-risk group had significantly higher TIDE scores (P < 0.001), leading to worse OS (P < 0.001) despite harboring more resting CD4^+^ T cells and M0 macrophages, whereas low-risk tumors were enriched in naïve B cells, plasma cells, and CD8^+^ T cells (all P < 0.05). Most immune checkpoint genes (PD-L1, CTLA-4, LAG-3) were differentially expressed between strata, reinforcing the model’s utility for predicting ICI response (94).

Bai et al. subsequently developed a five-lncRNA, m6A-centered risk model. Low-risk patients displayed markedly higher CD8^+^ T cell infiltration and elevated PD-1/CTLA-4 expression, translating to improved median OS (28.1 months vs. 14.7 months, P = 0.002) and a 2.3-fold higher ORR to pembrolizumab (95).

Apart from the m6A-centered lncRNA risk model, copper death-related lncRNAs were mined from 178 TCGA-PAAD and 95 CPTAC samples, yielding a six-gene signature (96). Low-risk tumors harbored 1.8-fold more CD8^+^ T cells and 2.1-fold higher PD-1 expression, indicating superior immunotherapy sensitivity, whereas high-risk tumors displayed distinct sensitivity profiles concerning gemcitabine and erlotinib.

Collectively, these lncRNA-based regulatory networks, which are methylation-driven, m6A-modified and copper-death-linked, function as robust, minimally invasive biomarkers that refine patient selection for ICIs, monitor therapeutic efficacy, and guide personalized combination strategies in PDAC.

### 3.2 Dynamic biomarkers in liquid biopsy

The advent of liquid biopsy has transformed the diagnosis and treatment of tumors. Liquid biopsy primarily consists of circulating tumor cells (CTCs), cell-free DNA (cfDNA), and ctDNA. CTCs are cells that detach from tumors and enter the bloodstream, whereas ctDNA consists of fragmented DNA shed by tumor cells into the circulation that can reveal genomic features. cfDNA includes DNA from both normal and tumor cells. ctDNA testing requires only blood collection and offers advantages such as real-time use, non-invasiveness, and repeatability. With technological advancements, detection methods such as ARMS, digital droplet PCR (ddPCR), and NGS are evolving, offering new tools that facilitate early tumor detection, continuous treatment monitoring, and resistance evaluation (97, 98). Recently, numerous studies have demonstrated significantly elevated cfDNA levels in the blood of patients with aggressive tumors, including pancreatic cancer, colorectal cancer, and melanoma (99–101). Previous studies extensively summarized the clinical applications of cfDNA in the diagnosis, prognosis, and assessment of surgical resectability in pancreatic cancer (102–104). An increasing amount of clinical evidence confirmed that dynamically monitored ctDNA, cfDNA, and CTCs have been employed as biomarkers for drug therapy in pancreatic cancer. This section will provide a detailed discussion of their roles in monitoring drug treatment in pancreatic cancer.

Dynamic monitoring of tumor-derived biomarkers enables real-time guidance of PDAC therapy. Wang et al. (105) demonstrated that the baseline CTC status predicts gemcitabine efficacy. Specifically, CTC-negative patients experienced significantly longer PFS and OS than CTC-positive patients. Extending this concept, Yu et al. (106) profiled circulating tumor-infiltrating cells (CTICs) from 77 patients with metastatic PDAC treated with FOLFIRINOX or gemcitabine/nab-paclitaxel. Chemosensitivity-predicted “effective” regimens yielded a median PFS of 7.8 months, significantly higher than that observed in the control group (4.2 months, HR = 0.35, P = 0.0002). Additionally, median OS was 21.0 months for patients who received predicted effective regimens, versus 9.7 months in the control group (HR = 0.40, P = 0.005), and accurately suggested active second-line options. Collectively, CTC/CTIC enumeration and molecular profiling provide actionable, minimally invasive biomarkers for tailoring chemotherapy sequences and improving outcomes in advanced PDAC.

Dynamic quantification of CTCs and ctDNA has evolved into a versatile, minimally invasive compass for tailoring PDAC therapy. As reported by Freed et al. (107), microfluidic isolation of epithelial (EpCAM^+^) and mesenchymal (FAPα^+^) CTCs in 31 niraparib-treated patients illustrated that a decreasing mesenchymal CTC/epithelial CTC ratio (Φ) identified 88% of responders and outperformed CA19-9 (*P* = 0.0093 vs. *P* = 0.033), correctly predicting the outcome in 72% of CA19–9 non-producers. Similarly, Lee et al. (108) prospectively followed 40 palliative chemotherapy-treated patients and found that total CTC and vimentin-positive CTC counts at 2 months were significantly linked to disease progression, as evidenced by PFS (P = 0.038) and response (*P* = 0.024 and 0.017, respectively). Building on these cellular data, Evrard et al. (109) demonstrated that ctDNA kinetics refine temporal assessment. Among 65 patients with KRAS-mutated cancer, high baseline cfDNA and persistent KRAS ctDNA levels at day 28 predicted poorer disease control, PFS, and OS. A composite score (cfDNA ≥ 30 ng/mL plus detectable KRAS ctDNA) best stratified response. Kitahata et al. (110) tracked 55 borderline-resectable patients in the neoadjuvant setting and observed that post-operative ctDNA positivity was linked to shortened median OS (not reached vs. 723 days, *P* = 0.0148), whereas combined ctDNA/CA19–9 monitoring further refined recurrence-free survival and OS. Hata et al. (111) noted that among 66 patients who underwent surgical resection, persistent post-surgical KRAS ctDNA levels were correlated with a higher liver-recurrence rate (*P* = 0.039). Moreover, Edland et al. (112) reported that longitudinal ctDNA detected progression 23 days earlier than imaging, underscoring its real-time sensitivity. Concerning precision therapy, Sudo et al. (113) screened 702 patients with advanced PDAC and identified BRCA1/2 and ATM mutations in 4.8% and 4.4% of patients, respectively, *via* ctDNA. These carriers achieved an ORR of 63.2%, versus 16.2% in wild-type patients, and patients with mutant cancer had longer PFS following platinum therapy (HR = 0.55, 95% CI = 0.32–0.93). Additionally, Pietrasz et al. (114) analyzed 354 patients with metastatic cancer using methylated HOXD8/POU4F1 ctDNA, with positivity (56.8%) independently predicting shorter PFS (5.3 months vs. 6.2 months) and OS (8.2 months vs. 12.6 months, HR = 1.62, *P* = 0.029) (115).

Collectively, these studies established CTC and ctDNA kinetics as real-time, quantitative biomarkers that both reflect the tumor burden and chemotherapy efficacy and stratify patients for targeted therapy, neoadjuvant therapy, and immunotherapy strategies, offering precision-guided, minimally invasive decision-making throughout the PDAC treatment continuum. In addition, dynamic monitoring of ctDNA and CTCs has displayed significant value for immunotherapy in other cancer types. For example, Gray et al. found that patients with initial ctDNA counts below the threshold of 10 copies/mL had a 5-fold higher likelihood of response to ipilimumab, nivolumab, or pembrolizumab than patients with high baseline ctDNA levels (95% CI = 1.8–13.8, P = 0.009). A decrease in the ctDNA concentration after 8 weeks of treatment was positively linked to treatment efficacy (115). Additionally, Xi et al. identified a robust link between peak BRAF ctDNA levels and the probability of objective response. Rapid declines in ctDNA levels were indicative of the potential for complete remission following treatment. Among the 13 patients who demonstrated this tendency, nine achieved complete remission, and four had a partial response (116). Ashida et al. also revealed that ctDNA levels dropped within 2–4 weeks in responding patients but remained elevated in non-responders (117). Goldberg et al. further confirmed this finding by examining 182 consecutive samples from 49 patients with metastatic NSCLC undergoing anti-PD-1 and/or anti-PD-L1 therapy. Patients with a ctDNA level reduction greater than 50% experienced longer-term benefits compared to those with less than 50% reduction (205.5 days vs. 69 days, *P* < 0.001) (118).

Although CTCs and ctDNA have been rarely reported in patients undergoing pancreatic cancer immunotherapy, lessons from their successful application in other malignancies indicate that they could become essential monitoring tools for immune-based treatment in PDAC. Pancreatic cancer immunotherapy faces formidable challenges, including a complex immune microenvironment, profound tumor heterogeneity, and a scarcity of reliable biomarkers. Serial quantification of CTCs and ctDNA can track the tumor burden in real time, identify patients most likely to benefit from immunotherapy, and guide adaptive treatment decisions. For example, early shifts in ctDNA counts can signal immunotherapy efficacy, enabling prompt regimen modification and sparing patients from unnecessary toxicities. Moreover, combined CTC and ctDNA profiling provides complementary insights that deepen our understanding of tumor dynamics and the overall impact of immunotherapeutic strategies.

### 3.3 Host–system biomarkers

#### 3.3.1 Gut microbiota characteristics

Mounting evidence positions the gut microbiome as a non-invasive, multifunctional biomarker for diagnosis, prognosis, and therapeutic guidance in PDAC. Kartal et al. (119) leveraged metagenomic and 16S rRNA sequencing in 136 Spanish (discovery) and 76 German (validation) stool samples, identifying a 27-species fecal signature that discriminated patients with PDAC from controls with an AUC of 0.84, which increased to 0.94 when serum CA19–9 was added. Crucially, the classifier retained robust accuracy across both early- and late-stage disease, underscoring its potential for population-scale screening. Expanding the geographic scope, Nagata et al. (120) profiled stool and saliva from Japanese, Spanish, and German cohorts (n ≈ 500) and revealed 30 gut and 18 oral microbial species that were consistently altered in PDAC. *Veillonella* and *Streptococcus* enrichment coupled with depletion of the anti-inflammatory microbe *Faecalibacterium prausnitzii* achieved AUCs of 0.74–0.83 across sites, demonstrating universal applicability and linking specific taxa to an elevated mortality risk. Metagenomic phage profiling further uncovered 58 viruses capable of infecting PDAC-associated bacteria, hinting at microbiome-directed therapeutic vectors. Beyond diagnostics, the microbiome influences drug efficacy. Geller et al. (121) analyzed 113 resected PDAC specimens, finding that 76% harbored Gammaproteobacteria capable of inactivating gemcitabine *via* cytidine deaminase. Co-administration of ciprofloxacin restored chemosensitivity *in vivo*. Metabolomic profiling indicated that chemotherapy responders had elevated fecal levels of indole-3-acetic acid, a microbiota-derived tryptophan metabolite with immunomodulatory properties. Finally, Pushalkar et al. (122) demonstrated that microbiota depletion reduced tolerogenic monocytes, enhanced CD8^+^ T cell infiltration, and increased PD-1 expression, thereby sensitizing PDAC to checkpoint blockade. Mechanistically, commensal microbes activated TLR-mediated pathways that fostered immune suppression. Antibiotic or probiotic modulation of these pathways reversed tumor tolerance and augmented anti-tumor immunity.

Collectively, these data establish the gut microbiota, through species abundance, metabolic output, and immune crosstalk, as a dynamic biomarker platform for PDAC diagnosis, prognosis, chemoresistance profiling, and immunotherapy optimization.

##### 3.3.2 Systemic inflammatory markers

##### 3.3.2.1 NLR

NLR has emerged as a simple, cost-effective barometer of systemic immunity that reliably tracks the response to ICB in pancreatic cancer. Shang et al. (123) retrospectively analyzed 120 patients with PDAC treated with ICB and found that an elevated baseline systemic immune–inflammation index (SII) increase the risk of death by 3.28-fold (*P* = 0.03), and each ≥20% increase in NLR during therapy further doubled the risk (HR = 2.21, *P* = 0.04). In the first-line ICB plus chemotherapy cohort, a higher baseline SII independently predicted shorter OS and PFS. A single-center study enrolled 98 patients with advanced PC who received PD-1 inhibitors (2015–2020) and stratified patients based on pre-treatment NLR ≥ 3 and lactate dehydrogenase (LDH) ≥ 250 U/L (124). The low-risk group (NLR < 3, LDH < 250 U/L) had a median OS of 44.2 months, versus only 6.4 months in the high-risk group (*P* < 0.01). Median PFS was 3.7 months in the low-risk group, versus 2.5 months in the high-risk group (*P* = 0.010), confirming the prognostic value of the composite biomarker (113). Rugambwa et al. (125) synthesized data from 40 studies (17 eligible for meta-analysis) and demonstrated that low NLR was correlated with a higher ORR and disease control rate, whereas high NLR predicted progression. A low platelet-to-lymphocyte ratio (PLR) mirrored these associations. Collectively, patients with lower NLR and PLR derived greater benefit from ICI therapy, whereas elevated ratios heralded treatment failure.

In summary, NLR, alone or combined with LDH and PLR, offers clinicians a readily available dynamic biomarker to gauge immunotherapy efficacy and refine prognosis in pancreatic cancer.

##### 3.3.2.2 IL-8

Serum IL-8 is emerging as a powerful, multipurpose biomarker in PDAC. Chen et al. (126) reported a median IL-8 level of 271.1 ± 187.7 ng mL^−1^ in patients with PDAC, markedly higher than that in patients with gastric cancer (41.8 ± 9.1 ng mL^−1^), colorectal cancer (78.7 ± 80.6 ng mL^−1^), or hepatocellular carcinoma (59.6 ± 19.8 ng mL^−1^), and found that high IL-8 levels were correlated with faster tumor growth and more aggressive biology. Independent research confirmed that elevated IL-6, IL-8, and IL-10 levels jointly predict poor prognosis in PDAC (127). Mechanistically, IL-8 recruits neutrophils and MDSCs, upregulates PD-L1 on tumor cells (128–130), and thereby fosters immune evasion. In the KPC mouse model, anti-PD-1 monotherapy was ineffective, whereas the addition of a CXCR2 inhibitor suppressed metastasis and significantly extended survival (131). Clinical translation is equally compelling, as high IL-8 expression has been linked to resistance to both chemotherapy and ICIs (132, 133). In melanoma and head-and-neck cohorts treated with nivolumab, pembrolizumab, or ipilimumab, elevated IL-8 expression predicted a lack of response, whereas decreased IL-8 expression signaled benefit (132, 134).

Collectively, these data establish IL-8 as a prognostic indicator with potential as a dynamic pharmacodynamic biomarker. While evidence from melanoma and head-and-neck cancers supports its use for treatment monitoring (132, 134), PDAC-specific prospective validation is required before IL-8 can be used to guide patient selection and on-treatment adjustments.

## 4 Emerging technology landscape

### 4.1 Background and rationale

The immune-escape web of PDAC is wired by a triple signature—scant neoantigens, an immunologically frigid core, and a stromal wall—so single-analyte biomarkers blink when asked to track its moving spatial–temporal patchwork. Over the past half-decade, the collision of single-cell multi-omics, attuned-sensor engineering, and AI has soldered liquid biopsy, live imaging, digital histology, and deep-omic layers into one self-updating circuit, handing clinicians a real-time feed on how immunotherapy momentum rises or stalls (135–137).

### 4.2 Single-cell multi-omics

Since 2019, successive upgrades of microfluidic systems—10x Genomics’ Next GEM (138) and BGI’s DNBelab C4 (139) among them—have made it routine to retrieve 1–2×10^5^ high-quality PDAC cells in a single run, with capture efficiencies steadily topping 90%. By blending scRNA-seq, scATAC-seq, scTCR-seq and CITE-seq (RNA plus surface proteomics) within the same tissue specimen, laboratories can now profile epigenome, transcriptome, immune receptors and membrane proteins in one integrated workflow (140, 141).

In 2024, Li et al. (137) profiled freshly resected tumors from 31 stage I–IV PDAC patients using paired scRNA-seq and scATAC-seq, averaging 78,632 cells per case and 1,426 genes per cell. Multimodal integration uncovered a previously undescribed complement-secreting cancer-associated-fiber population (csCAF) that expands with stage and is defined by high C3, CFD and CSF1 expression. Functional work showed that csCAF drives CD8^+^ T-cell exhaustion through the CSF1-CSF1R axis; daily BLZ945 (50 mg kg^-^¹ i.p. for two weeks) reduced csCAF abundance (P = 0.002), restored IFN-γ and TNF-α production by intratumoral CD8^+^ T cells, and restrained tumor growth by 62% in KPC mice. One year later, Klein et al. (136) combined Visium HD (2-µm spots, near-single-cell resolution) with 10x scRNA-seq on 31 stage I–IV surgical samples (again ~78,000 cells each) and revealed that basal-like and classical sub-clones share the same lesion but occupy distinct micro-niches: basal-like cells dominate the hypoxic core, whereas classical cells cluster at the invasive edge. This spatial co-existence is maintained by the JUNB/AP1 axis; genetic or pharmacologic blockade of JUNB (shRNA) or AP1 (SR 11302) up-regulated tumoral HLA-I and CD80, boosted dendritic-cell cross-presentation, and lifted anti-PD-1 response rates from 12% to 58% in the KPC model (P = 0.003).

Looking ahead, the field is expected to advance along three tracks (142–144): (i) combinatorial-index microfluidics that capture scRNA, scATAC, scTCR and surface proteome in one run at >200,000-cell throughput and 40% lower cost; (ii) a 26-centre PancCellAtlas that already hosts 1,000 four-omics samples to serve as a standard training repository for AI tools; and (iii) MRI-guided, magnetically steered 18-gauge needles for real-time, spatially precise intra-operative sampling.

### 4.3 Ultrasensitive detection platforms

PDAC plasma typically contains <0.1% ctDNA, and KRAS mutant VAF can drop to 0.01%, so conventional NGS misses more than half of cases: the tumor bulk is small, encased in dense stroma, mutations are scattered across sub-clones, and normal cfDNA background reaches 10³–10^4^ GE/mL while mutant molecules contribute <10 GE/mL, pushing the required limit of detection to attomolar or single-copy levels (2, 114, 145).

In 2024 Batool et al. (146) combined a gold nano-island SPR chip with a PD-L1 aptamer, completing serum PD-L1 read-out in 30 min with an LOD of 0.3 pg/mL; a ≥20% drop in PD-L1 at day 14 of immunotherapy matched objective response in 92% of cases. The same year Shah et al. (147) developed a CD8-targeted short-wave infrared probe that yielded 6.8-fold higher signal in “hot” tumors and imaged CTL infiltration at 250 µm resolution with 200 ms exposure. In 2025 Hu et al. (148) introduced the NAPTUNE dual-enzyme cascade platform, detecting 1 aM circRNA/miRNA in 45 min without amplification; KRAS G12D remained reliably positive at 0.0003% VAF, giving 95% sensitivity and 100% specificity.

Over the next 3–5 years a 23 mm×23 mm all-in-one microfluidic cartridge will simultaneously quantify ctDNA, methylation, exosomal miRNA and IL-8 within 30 min, with an LOD of 1 copy µL^-^¹ for ctDNA; NIST standard SRM-2367a (0.1–10 aM KRAS G12D) has already been used to calibrate NAPTUNE, and a five-center, 240-sample blinded validation showed <8% CV and 97.5% positive agreement, paving the way for a forthcoming 15-centre multicenter study (148, 149).

### 4.4 AI-integrated predictive models

With PDAC immunotherapy still delivering objective response rates below 10%, a non-invasive tool to single out likely beneficiaries has become urgent. Between 2022 and 2025 Bian’s group pushed a “dual-mode” radiology-plus-epigenomics model through three rounds of refinement: an initial contrast-enhanced CT radiomics/XGBoost classifier reached an AUC of 0.79 for CD8^+^ TIL status (150) (10), the same pipeline proved stable on non-contrast MRI (AUC 0.76) (151). In parallel, the SCORPIO ensemble trained on routine blood panels from 1–628 patients across 17 tumor types and locked its performance in 4–447 external cases; the gradient-boosting model predicted immunotherapy overall survival with an AUC of 0.76, outperforming both TMB (0.58) and PD-L1 IHC (0.54), and defined a low-risk group whose mortality hazard was only 0.16 that of the high-risk group and whose objective benefit rate was 65% versus 33% (P < 3×10^-^¹¹) (152). Also in 2025, Zang et al. (153) unveiled a Transformer-based, multi-modal monitoring system that simultaneously ingests bi-weekly CA19–9 slope, ctDNA decay constant k, IL-8 percentage change and CT-derived TIL radiomic score; it achieved an AUC of 0.89 for predicting six-month objective response in 412 advanced NSCLC patients and is now being trialed in PDAC under protocol NCT05678921 (n = 300, primary endpoint 24-week disease-control rate, read-out expected Q4 2026). The same consortium is preparing “PDAC-IMMUNO-AI”, a federated-learning hub that will pool >5–000 multi-omics cases from 20 centers worldwide without moving raw data, embed SHAP/Grad-CAM explainability to quantify the contribution of each imaging, methylation or ctDNA feature (152), and deploy an on-line incremental algorithm that refreshes model weights within five minutes as new liquid-biopsy data arrive (152), keeping predictive accuracy in step with each patient’s treatment course.

## 5 Challenges and future directions

### 5.1 Bottlenecks in translational applications

#### 5.1.1 The challenge of spatial heterogeneity

PDAC is notorious for its spatial heterogeneity. Specifically, basal-like (BL) and classical (CLA) subtypes coexist within the same tumor, complicating single-site biopsies. Klein et al. (136) reported that intrinsic AP1 transcription factors (JUNB/cJUN) and an extrinsic tumor immune microenvironment cooperate to maintain this mixture. JUNB/AP1- and HDAC-driven networks restrict macrophage infiltration, and these macrophages subsequently disrupt CLA epithelial integrity and propagate the BL phenotype, fostering an immunosuppressive milieu and treatment resistance. To capture this complexity, spatial phenotyping signatures derived from multiplex immunohistochemistry (mIHC) or multiplex immunofluorescence (mIF) quantify cell density and tumor–immune interactions across entire tissue sections. Locke et al. (3) and Hoyt et al. (154) noted that mIF overcomes spectral overlap inherent to bright-field dyes, but clinical deployment is restricted by complexity, standardization, autofluorescence in FFPE tissues, and rigorous quality control. Although AI-enhanced image analysis is routine for conventional IHC, it remains underutilized for the richer mIHC/mIF datasets. Continued refinement of protocols, calibration standards and regulatory-grade software is essential to translate these high-resolution spatial maps into everyday clinical decision-making.

#### 5.1.2 Insufficient monitoring of dynamic evolution

The TME of PDAC is dynamic, as its cellular composition and molecular landscape evolve markedly as the disease advances and, even more dramatically, during therapy. This “biomarker drift” can undermine real-time monitoring of treatment efficacy and compromise prognostication. Gao et al. demonstrated that standard chemotherapy profoundly rewires the TME. In addition to its direct cytotoxicity, the regimen triggered a notable rise in myeloid marker lysozyme (LYZ) counts within malignant cells, re-shaped antigen presentation networks, and shifted poliovirus receptor (PVR) signaling from T cell TIGIT to myeloid cell CD226 engagement. These alterations jointly foster an immunosuppressive milieu that can seed resistance and eventual relapse (155). Complementing these functional insights, Li et al. constructed a 40,542-cell atlas across 31 PDAC samples spanning stages I–IV. They documented stage-dependent remodeling of the ductal, immune, and stromal compartments. In particular, complement-secreting CAFs accumulated with advancing stage, whereas pancreatic stellate cell counts dwindled, underscoring the dynamic nature of stromal heterogeneity (137).

To capture such fluctuations in real time, high-frequency liquid biopsy coupled with rigorously timed sampling has emerged as a practical countermeasure. In a prospective cohort of 204 patients receiving 260 systemic therapies, serial plasma was collected before treatment, during treatment, and at the first re-assessment. Gene-informed ddPCR was performed to quantify ctDNA levels. Progressors (defined radiologically) exhibited higher ctDNA detection rates at every time point (*P* < 0.009) and shorter median time-to-treatment failure (*P* ≤ 0.001). Rising ctDNA levels heralded progression in 73% of patients with a median lead time of 23 days (156). Similar observations were reported by Ko et al. in advanced esophageal squamous-cell carcinoma, in which serial liquid biopsies furnished complementary predictive and prognostic information beyond imaging (157).

Collectively, these data affirm that the PDAC TME is in constant flux. Only dynamic, minimally invasive biomarker monitoring can accurately monitor this evolution and guide precision oncology decisions.

### 5.2 Multimodal integration: the way forward

The use of single biomarkers in predicting the response to immunotherapy in pancreatic cancer has significant limitations. For example, PD-L1 expression varies significantly across different tumor types and individuals, and its expression can be influenced by multiple factors, such as the TME and therapeutic interventions. TMB, although displaying some predictive value in certain cancer types, faces challenges in its application in pancreatic cancer, such as the lack of standardized detection methods and thresholds. ctDNA can reflect changes in the tumor burden in real time, but its specificity and sensitivity in predicting immunotherapy response require further improvement.

To overcome these limitations, future research must construct multidimensional integrated models that consider several biomarkers and clinical characteristics to improve the accuracy and reliability of predictions. Multidimensional integrated models can more comprehensively reflect the biological characteristics of tumors by considering multiple factors, including tumor cells, immune cells, stromal cells, and the extracellular matrix. By integrating multiple biomarkers, these models can better capture the dynamic changes of tumors during treatment, thereby more accurately assessing treatment responses. Moreover, multidimensional integrated models can provide personalized treatment recommendations based on individual patient characteristics, thereby improving treatment outcomes.

**Figure 3:**
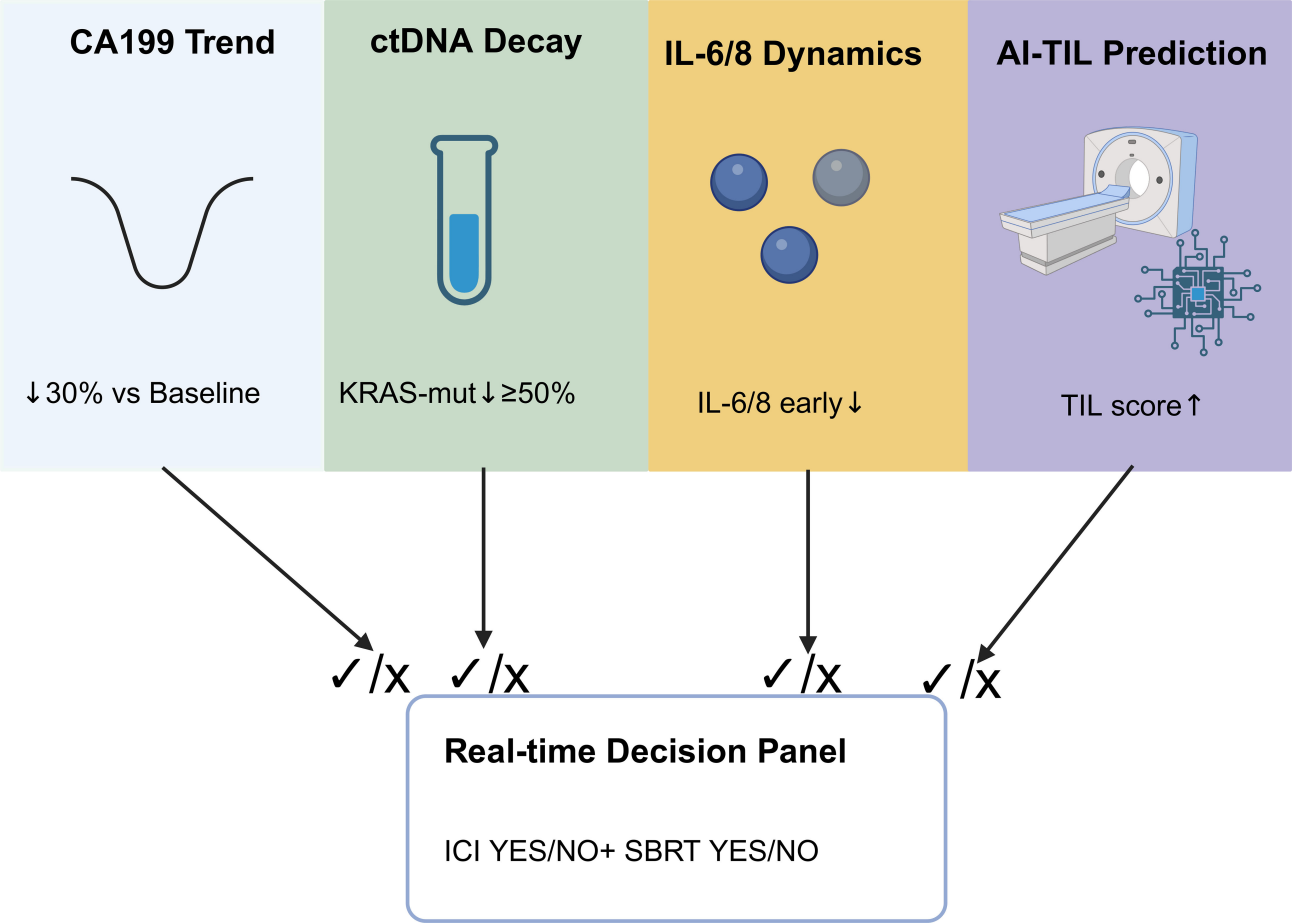
Multiplex biomarker dashboard for precision immunotherapy guidance in PDAC.

Multimodal integrated models hold significant advantages in enhancing immunotherapy outcomes in pancreatic cancer, overcoming the limitations of single biomarkers and providing more comprehensive and accurate predictions. Future research should further optimize model construction methods, validate the clinical applicability of these models, and promote the widespread application of multimodal integrated models to optimize immunotherapy outcomes in pancreatic cancer, thereby permitting more precise treatment strategies and better patient outcomes. As illustrated in **Figure 3**, integrating CA19–9 kinetics, ctDNA decay, IL-6/8 dynamics, and AI-based TIL prediction into a real-time decision dashboard enables precision navigation of immunotherapy for PDAC.

Despite the significant advantages of multimodal integrated models, which can overcome the limitations of single biomarkers and provide more comprehensive and accurate predictions, in pancreatic cancer immunotherapy, current research primarily focuses on biomarker integration and the application of AI. However, nanotechnology, as an emerging cutting-edge technology, also has great potential in detecting biomarkers and monitoring immunotherapy efficacy. For example, nanotechnology has demonstrated considerable potential in monitoring tumor immunotherapy responses. Shah et al. (147) developed shortwave infrared-emitting nanoprobes that can specifically target CD8^+^ cytotoxic T cells, facilitating *in vivo* imaging in a breast cancer mouse model with high sensitivity. Whether this approach can effectively penetrate the desmoplastic, hypoxic PDAC microenvironment to image sparsely distributed CD8+ T cells requires dedicated PDAC-specific validation. Batool et al. (146) utilized a nanoplasmonic sensor to detect changes in serum PD-L1 and cytokine levels (*e.g.*, IFN-γ, IL-2) within 1–2 weeks after treatment in clinical samples, with sensitivity reaching the picomolar level, highly consistent with imaging results. Chang et al. (158) combined fluorescence and photoacoustic imaging in the NanoTrackThera platform to monitor the efficacy of tumor immunotherapy and photothermal therapy in real time, providing strong support for precision immunotherapy. The introduction of nanotechnology can further refine multimodal integrated models, offering more comprehensive and precise solutions for pancreatic cancer immunotherapy and promoting the development of precision immunotherapy.

## 6 Conclusion

Research on biomarkers for immunotherapy in pancreatic cancer has evolved from single-molecule approaches to multimodal dynamic integration. Traditional biomarkers (*e.g.*, CA19-9, MSI) hold value in specific contexts, but emerging biomarkers, including H3K18la, MED12, and dynamic ctDNA clearance rates, offer new perspectives for overcoming immunotherapy resistance. With the development of spatial multiomics, single-cell technologies, and AI, future biomarker models will integrate the tumor’s intrinsic characteristics (*e.g.*, genomic, epigenetic), microenvironmental status (*e.g.*, immune, metabolic, microbiota), and host factors (*e.g.*, inflammation, microbiome), ultimately converting “cold tumors” into “hot tumors.” In this process, interdisciplinary collaboration, standardized detection, and prospective clinical trial validation will be the key safeguards for promoting the clinical translation of biomarkers.

Despite significant advancements in research on biomarkers for pancreatic cancer immunotherapy in recent years, certain limitations remain. The primary issue is that most research has focused on single biomarkers or combinations of a few biomarkers, lacking large-scale clinical validation. Although these studies provided valuable insights, in practical applications, single biomarkers often fail to comprehensively reflect the complexity and heterogeneity of tumors, limiting their value in clinical decision-making.

To address these limitations, future research should focus on several directions.

Multicenter, prospective clinical trials: Multicenter, prospective clinical trials are recommended to validate the clinical applicability of multimodal integrated models. These trials should encompass a variety of biomarkers and clinical characteristics to ensure the accuracy and reliability of the models.

Standardization of detection methods: Further optimization and standardization of biomarker detection methods are needed to ensure that the results are consistent and comparable across different laboratories and clinical centers, facilitating more reliable research and clinical practice.

Interdisciplinary collaboration: Strengthening interdisciplinary collaboration and integrating expertise from multiple fields, including oncology, immunology, bioinformatics, and clinical medicine, will drive in-depth biomarker research.

Dynamic monitoring and **real-time** feedback: The development of technologies and methods capable of continuously tracking tumor dynamics in real time is essential to enable timely adjustments of treatment plans and enhance therapeutic outcomes.

In summary, although progress has been made in the identification of biomarkers for pancreatic cancer immunotherapy, additional efforts are needed concerning the construction and validation of multimodal integrated models in the future. Through interdisciplinary collaboration and large-scale clinical trials, the precision and effectiveness of pancreatic cancer immunotherapy can be further improved, ultimately leading to better patient prognosis.

## Glossary

PDAC: Pancreatic ductal adenocarcinoma
ICB: immune checkpoint blockade
NSCLC: non-small cell lung cancer
PD-1: programmed cell death protein 1
PD-L1: programmed death ligand 1
CTLA-4: lymphocyte-associated protein 4
TME: tumor microenvironment
TAMs: tumor-associated macrophages
TGF-β: transforming growth factor-β
CAFs: cancer-associated fibroblasts
Tregs: regulatory T cells
TMB: tumor mutational burden
MSI: microsatellite instability
ctDNA: Circulating tumor DNA
AI: artificial intelligence OS, overall survival
HR: hazard ratio
NLR: neutrophil-to-lymphocyte ratio
TPS: tumor proportion score
IHC: Immunohistochemistry
ICIs: immune checkpoint inhibitors
dMMR: deficient mismatch repair
RNA-seq: RNA sequencing
MPI: metabolic prognostic index
MEMG: mitochondrial energy metabolism-related gene
PTX3: pentrexin 3
AUC: area under the curve
H3K18la: lactate and its associated histone lactylation modification
MED12: Mediator subunit 12
lncRNA: long non-coding RNA
CTCs: circulating tumor cells
cfDNA: cell-free DNA
ddPCR: digital droplet PCR
CTICs: circulating tumor-infiltrating cells
LDH: lactate dehydrogenase
PLR: platelet-to-lymphocyte ratio
mIHC: multiplex immunohistochemistry
mIF: multiplex immunofluorescence
NAPTUNE: nucleic acids and protein testing via ultrasensitive nuclease escalation
TIL: tumor-infiltrating lymphocyte
RF: random forest
